# Insights Gained and Future Outlook From scRNAseq Studies in Autoimmune Rheumatic Diseases

**DOI:** 10.3389/fimmu.2022.849050

**Published:** 2022-02-17

**Authors:** Zihan Zheng, Ling Chang, Jingyi Li, Yuzhang Wu, Guangxing Chen, Liyun Zou

**Affiliations:** ^1^ Institute of Immunology, Army Medical University, Chongqing, China; ^2^ Department of Autoimmune Diseases, Chongqing International Institute for Immunology, Chongqing, China; ^3^ Department of Rheumatology and Immunology, First Affiliated Hospital (Southwest Hospital) of Army Medical University, Chongqing, China; ^4^ Center for Joint Surgery, First Affiliated Hospital (Southwest Hospital) of Army Medical University, Chongqing, China

**Keywords:** rheumatic and muscoluskeletal disease, single-cell ‘omics, autoreactive antibodies, single-cell RNA sequencing, trajectory tracking, rheumathoid arthritis, systemic lupus - erythematosus, Sjogren’ s syndrome

## Abstract

Autoimmune rheumatic diseases have a major impact on public health as one of the most common morbidities, and many of these disorders involve both local and systemic manifestations with severe consequences for patient health and quality of life. However, treatment options for many of these diseases remain inadequate for a substantial portion of patients, and progress in developing novel therapeutics has been slow. This lack of progress can be largely attributed to an insufficient understanding of the complex mechanisms driving pathogenesis. Recently, the emergence of single-cell RNA sequencing (scRNAseq) has offered a powerful new tool for interrogating rheumatic diseases, with the potential to assess biological heterogeneity and individual cell function in rheumatic diseases. In this review, we discuss the major insights gained from current scRNAseq interrogations of human rheumatic diseases. We highlight novel cell populations and key molecular signatures uncovered, and also raise a number of hypotheses for follow-up study that may be of interest to the field. We also provide an outlook into two emerging single-cell technologies (repertoire sequencing and spatial transcriptomics) that have yet to be utilized in the field of rheumatic diseases, but which offer immense potential in expanding our understanding of immune and stromal cell behavior. We hope that scRNAseq may serve as a wellspring for the generation and interrogation of novel hypotheses regarding autoreactive lymphocytes and tissue infiltration patterns, and help uncover novel avenues for therapeutic development.

## Introduction

Autoimmune rheumatic diseases have a major impact on public health as one of the most common morbidities, affecting some 3% of the general population (1). Many of these disorders involve both local and systemic manifestations in multiple organs, leading to severe health consequences and compromising patient quality of life. Treatment options for these disorders are largely limited to immunosuppressants, in the form of conventional synthetic drugs such as methotrexate, or monoclonal antibodies against specific targets (biologics). While many of these treatments are effective at first pass in alleviating patient symptoms, a substantial portion of patients are refractory towards treatment (2). Others may still suffer from periodic flares in disease activity, and/or loss of drug efficacy over time.

However, the development of new therapeutics for improved treatment has been painstakingly slow, with no treatment having been approved for primary Sjogren’s syndrome, and very few for systemic lupus erythematosus, leading to major difficulties in prescribing more precise clinical guidelines tailored for individual patients (3, 4). This lack of progress can be largely attributed to an insufficient understanding of the complex mechanisms driving pathogenesis. In particular, the what/where/how questions regarding the cell types causing disease progression have only been incompletely addressed, and higher-level knowledge of how disparate cell types may interact with each other and their environment in these diseases is lacking. Attempting to answer these big-picture questions using older low-throughput technologies has proven to be a laborious endeavor. Fortunately, the emergence of new high-throughput technologies for assessing biological heterogeneity may provide fruitful approaches for tackling these questions.

Since its advent from relatively low-throughput applicability, single-cell RNA sequencing (scRNAseq) has evolved into a powerful tool for assessing biological heterogeneity and individual cell function in complex environments at extremely high throughput (5, 6). Unlike previous bulk sequencing studies where unique characteristics of a particular population of cells might be hidden by complex changes in the overall average, scRNAseq can be used to efficiently classify changes in individual cell types with simultaneous knowledge of global and local structure. As such, scRNAseq can identify rare populations and their functional characteristics in an unbiased manner in physiological and pathological conditions. Furthermore, trajectory inference based on minute transcriptome changes can be used to accurately predict differentiation and/or developmental hierarchies within and between cell populations (7). Novel bioinformatics tools have also enabled more detailed dissection of the pathways and molecules contributing to trajectory progression (8), as well as communications patterns between cells (9). In the context of rheumatic diseases where the overall composition, differential hierarchies, and functional characteristics of cell types contributing to disease remains incompletely understood, scRNAseq thus holds considerable promise for as a key method for unlocking novel, unbiased insights. These insights may further serve as foundational resources for uncovering new avenues for translational research and therapeutic development.

In this review, we first summarize major findings obtained from current scRNAseq studies in rheumatoid arthritis, systemic lupus erythematosus, and primary Sjogren’s syndrome. We also highlight a number of provocative questions raised by the results obtained from these studies, and which may be of relevance towards furthering current knowledge in the field. We further discuss the potential value for integrating other forms of single-cell omics data into these mapping studies and beyond, to preview some forms of potential knowledge to come from future studies.

## Rheumatoid Arthritis

Rheumatoid arthritis (RA) is an autoimmune disease characterized by erosion of the protective collagen layer in joints across the body under inflammatory stress (10). In a significant majority of cases, this stress is directly provided by the infiltration of large numbers of immune cells into the synovial membrane, although a leuckocyte-poor presentation of RA has also been described (11). Infiltrating immune cells have been demonstrated to secrete a wide range of pro-inflammatory cytokines to stimulate local inflammation and osteoclast differentiation, and may also help to promote excessive proliferation of fibroblast-like synoviocytes (FLS) (12). The resulting increase in synovial fluid production is a key driver of the joint swelling observed in RA, and may also promote the formation of a complex joint microenvironment. Dissecting the full complexity of this microenvironment has proven to be difficult. However, the advent of scRNAseq has allowed for more comprehensive census of the cell types present and their functional states, as reviewed below.

A number of efforts have been made to generate a cellular atlas of rheumatoid arthritis in both peripheral circulation and in afflicted joints. Two early single-cell mapping studies using a homebrew microfluidic system (13) or the plate-based CEL-seq2 method (14, 15) both reported striking heterogeneity among synovial FLS, characterizing a novel population expressing Thy1 (CD90) and lacking expression of CD55. In contrast to the CD55+ FLS distributed in the synovial membrane lining, Thy1+ FLS localize more deeply within synovial tissue. These Thy1+ FLSs also appear to express key chemokines, such as CXCL12, and might contribute to the recruitment of immune cells to synovial tissue. Unfortunately, the relative proliferative potential of these FLS subtypes has not been examined, and it is unclear if these FLS will become a dominant population as RA progresses. It is also not fully clear if these FLS retain the ability to produce synovial fluid, and/or if the composition of the fluid produced is distinct between subtypes.

Interestingly, a portion Thy1+ FLS may also express the MHC-II molecule *HLA-DRA*. These results raise an interesting possibility that differentiation of synovial FLS towards a pro-inflammatory state may occur as an early event during RA pathogenesis, with this conversion potentially contributing to the recruitment of immune cells to synovial tissue. The potential of synovial FLS derived from RA patients to act as additional antigen-presenting cells to activate T cells has been previously demonstrated in several reports (16, 17). However, these previous reports all included an additional interferon-gamma sensitization step, leading to some questions regarding the true relevance of these *in vitro* assays. In light of the scRNAseq results, it may be interesting to re-evaluate the antigen-presentation potential of synovial FLS by focusing only on the Thy1+ subpopulation. If these FLS are indeed capable of antigen presentation without the need for additional cytokine stimulus, and can easily localize near infiltrating T cells in synovial tissue, targeting this population may prove to be essential for preventing further relapse in RA. A preliminary clinical trial (TRAFIC, ISRCTN36667085) has already been initiated to target this FLS in patients refractory to methotrexate, and results from these studies may yield additional insights into their function (18).

Recently, another mapping-focused study has been performed using samples from patients with or without anti-citrullinated peptide antibodies (ACPA, a type of autoantibody observed in roughly two-thirds of patients with established RA) in a cohort with minimal treatment intervention (19). Interestingly, the study reported a significant decrease in the expression of the MHC-II molecule *HLA-DRB5* on a portion of plasma cells in the peripheral blood of ACPA+ patients compared to both ACPA- counterparts and healthy controls, and with remarkable similarity to naïve B cells. As these HLA-DRB5+ plasma cells do not appear in the concurrently sampled synovial specimens, it is possible that this population may represent a protective phenotype against RA, or might otherwise be composed of B cells with high affinity for antigens unrelated to RA progression. Variation in MHC-II genes has been previously reported to be significantly associated with RA from gene association studies, with variation in *HLA-DRB1* having been long identified to be correlated with levels of ACPA, a finding reproduced in a number of cohorts using different technologies (20, 21). It may be interesting to evaluate if *HLA-DRB5* may also be a concurrent or independent risk allele for ACPA levels, and if expression changes in *HLA-DRB5* occur independently/concurrently with expression changes in other MHC-II genes and/or molecules involved in antigen presentation and co-stimulation.

At the same time, the study also noted a substantial increase in the expression of chemokines from the CCL family in both dendritic cells and macrophages of ACPA- patients compared to ACPA+ counterparts. If these increases are indeed independent of current patient disease severity, this result may suggest an alternative, more innate-centric model for disease progression in ACPA- patients, compared to a more lymphocyte-driven reaction in ACPA+ counterparts. Such a divergence in mechanism may partly explain clinically observed differences in disease progression (22). Further investigations may help to shed light on these possibilities.

Other recent studies have focused on a specific population of cells to investigate them in greater detail. For instance, a study focused on the macrophage compartment in RA synovial tissue emphasized the existence of CD206+MerTK+ macrophages with a unique regulatory signature in RA patients experiencing clinical remission, and correlated with remission maintenance (23). This population matches up well with a population of residential joint-protective macrophages identified in mice (24). Since CD206+ has been traditionally used to identify regulatory M2 macrophages in multiple contexts, and stimulation of MerTK signaling has been shown to favor M2 differentiation, these results are in one respect unsurprising (25, 26). Interestingly however, the study also indicated that a primary ligand for MerTK, Gas6, was abundantly produced by sublining Thy1+ FLS, and that this production occurred simultaneously with that of the chemokine CXCL14 in patients with active disease and experiencing remission. Notably, MerTK+ macrophages were largely located in the lining layer, and were thus not in direct contact with the Thy1+ FLS, while CD68+MerTK- macrophages were present in the sublining. This latter result suggests that apparently pro-inflammatory FLS may also serve as a key driver of a homeostatic regulatory circuit by promoting differentiation of M1 macrophages towards M2 phenotypes. Additional work on this homeostatic circuit and the identification of biological pathways to promote M2 conversion and Thy1+ FLS production of Gas6 may thus be of considerable therapeutic interest.

## Systemic Lupus Erythematosus

Systemic lupus erythematosus (SLE) is a severe autoimmune disorder typically involving systemic manifestations capable of causing major, and oftentimes chronic, organ and tissue damage (27, 28). The clinical progression of SLE frequently involves repeated series of flares in disease activity followed by period of remission, for reasons that remain incompletely understood. From an immunological standpoint, it is commonly appreciated that autoantigens derived from cell nucleus components (both proteins and double-stranded DNA) are the key drivers of disease, and high titres of circulating anti-nuclear antibodies (ANA) typically correspond with elevated disease activity (29). These autoantibodies have been shown to form large deposits in the kidneys and other organs, and are a key predictor of renal injury, presumably *via* antibody-mediated damage (30, 31). As such, the search for novel therapeutics against SLE has emphasized the identification of drugs capable of restraining B cell activity and antibody production. However, by virtue of its systemic nature, a large range of immune cell types are likely to be involved in SLE disease progression.

In order to map this systemic disease, large cohort studies have been conducted to characterize the immune populations present in peripheral circulation of SLE patients. The first of these studies focused on identifying populations of immune cells especially responsive to interferon stimulation (32). These analyses led to the identification of IL-1β+ monocytes, CD11c+ B cells, and FCGR3A+ NK cells as expressing interferon-stimulated genes (ISGs) at high levels. These increases appeared to be systemic among the patients sequenced, and some of these populations appear to be enriched among patients with higher disease activity scores, although the actual correlations are not fully clear. Notably, despite longstanding concerns regarding patient heterogeneity in rheumatic diseases being a major confounding factor, the actual heterogeneity in regards to cell type changes appears to be quite small, and at a level controllable by batch normalization. More importantly, these data emphasize the important influence of interferon signaling in the context SLE, and demonstrate that pervasive interferon signaling may significantly reshape the differentiation processes of circulating immune cell populations. Of note, a similar ISG signature has also been observed in low-density granulocytes (33) and in dendritic cells (34), further demonstrating its reach.

While a large number of interferon genes exist, there has long been evidence suggesting that IFNα may be the one most strongly correlated with disease activity in SLE, and treatment with IFNα may provoke SLE-like side effects in some patients (35, 36). Intriguingly, a monoclonal antibody against the IFNα receptor has also emerged in early clinical trials as a candidate with significant promise in patients with moderate-to-severe SLE, although results in phase III have not yet been clarified due to differing choices in primary endpoint designation between two trials (NCT01438489, NCT02446912, NCT02446899), with significance relative to placebo being found using one metric, but not the other (37–39). Further investigations may help clarify if the abundance of ISGhi immune cells is indeed predominantly caused by signaling from IFNα, and the suitability of targeting this pathway for treating a broad population of SLE patients. For instance, one possibility may be that patients with the highest ISG activity may be more sensitive to intervention through IFNα compared to patients with lower ISG activity.

At the same time, additional explorations of other biological pathways and processes that are highly correlated with progression towards ISGhi signatures may provide additional insights into the mechanisms by which these cell populations exert pathological functions. In particular, it is unclear if continued expression and stimulus of interferon receptors is required for the maintenance of these ISGhi cell states, or if they represent a more stable end-state. Trajectory analyses that reconstruct the differentiation hierarchies between cell states may be particularly useful in this context for identifying potential novel forks in differentiation caused by the elevated levels of interferon observed. The inclusion of additional data from currently unpublished efforts to study signatures in patients with acute flares and with other unique pathological features may help to further clarify this possibility.

Beyond the peripheral transcriptome, other studies have focused on evaluating regional differences brought about by SLE pathology in the kidneys and skin. By isolating immune cells from renal biopsies, one study was able to capture a complex landscape of renal-infiltrating immune cells broadly similar to the peripheral atlas, including several populations of T and B cells defined by an ISG signature (40). Part of this signature could also be recapitulated in urine, raising the potential for a non-invasive method for assessing lupus nephritis (41). Similarly, a study of lesional and non-lesional skin from SLE patients also reported increases in ISG signature in T cells (42).

Interestingly, the renal landscape also recovered an increase in ISG signature in tissue resident macrophages expressing the transcription factor *BLHLE41*, consistent with the hypothesis that the function and transcriptome of residential immune cells may also be significantly altered as a result of interferon signaling in SLE. However, substantial numbers of T and B cells have also been demonstrated to be present in the kidneys under physiological conditions (43). It is unclear how these other residential populations might be impacted by interferon signaling, or if they might be displaced by the large numbers of infiltrating cells. After all, these T and B cell populations also include significant proportions of naïve B cells as well as T cells with a follicular-helper phenotype, raising a possibility that T and B cells may even localize together into germinal center-like structures in the kidney. While histological studies of lupus nephritis have previously found associations between these structures and worsened clinical outcomes, it remains unclear if these structures can indeed facilitate affinity maturation of B cells. Additional immune repertoire focused sequencing may help to clarify this question.

## Primary Sjogren’s Syndrome

Primary Sjogren’s syndrome (pSS) is characterized by excessive dryness at mucosal membranes due to insufficient fluid production from secretory glands (particularly salivary and lacrimal glands) (44). Histological examination of these glands in patients with pSS will typically identify immune foci formed by infiltrating T and B cells, and these cell types are generally understood to contribute significantly to disease pathogenesis. A significant portion of patients with pSS will also experience chronic fatigue, and symptomatic manifestations in a number of organs have been reported, although the underlying mechanisms driving systemic effects and possible disease subtypes are unknown at this time (45). pSS has also proven to be especially difficult to treat, with current therapy often limited to alleviation of symptoms (such as artificial tears for sicca) (46). This intractability is due to traditional immunosuppressants failing to demonstrate durable clinical efficacy either alone or in combination in larger randomized clinical trials. However, a number of early stage trials evaluating novel compounds and/or biologics have yielded some promising early results. For instance, sustained reductions in disease activity through 24 weeks *via* treatment with an antibody targeting the BAFF receptor has been recently reported in a phase 2b trial (NCT02962895) (47). Further research is urgently needed to clarify if these biologics and/or other drugs do in fact represent viable therapeutic options for pSS.

Thus far, one preliminary scRNAseq study has been performed examining the peripheral blood of patients with pSS as compared with healthy controls (48). The authors reported an increase in CD4+ T cells with a potentially cytotoxic phenotype in pSS patients, with this population appearing to be transcriptionally similar to CD8+ CTLs from the dimension reduction provided. At the same time, follow-up flow cytometry data presented raises confusion as to the expected percentages of this population within CD4 T cells. Interestingly, earlier histological examinations of minor salivary glands in pSS patients have reported the existence of high percentages of CD4 CTLs in SS lesions (49). In addition, CD4 CTLs in other contexts have been demonstrated to be capable of directly killing target cells based on recognition mediated by MHC-II (in contrast to MHC-I recognition in conventional CD8+ CTLs) (50). It may be interesting to investigate if peripheral blood CD4 CTLs in pSS are clonally and functionally associated with the lesional CD4 CTLs seen in salivary glands, and if these cells can perform MHC-II mediated targeted killing of glandular exocrine cells.

The establishment of a clonal relationship between peripheral and gland-infiltrating T cell populations may be especially important in the context of pSS due to the complexity of the peripheral signature of pSS. A number of large-cohort studies using bulk RNAseq of peripheral blood have found remarkably few differences between the pSS patients and healthy controls, with most of the variation being concentrated in the interferon signaling pathways (51–53). As such, one interpretation may be that a few interferon-sensitive populations might be substantially related to disease progression. It is unclear if interferon signaling may also modulate the presence and/or function of CD4 CTLs, or of the other populations observed. Two preliminary single-cell repertoire sequencing studies of gland-infiltrating T cells has already been performed, and have reported clonal expansion of select T helper populations (54, 55). Additional work, ideally with concurrent sampling of multiple afflicted glands, will be necessary to further clarify the immune cell populations contributing to pSS pathogenesis, and potentially identify novel therapeutic strategies for this condition.

## Summary and Outlook From Current scRNAseq Studies

Atlas-focused mapping studies have been conducted in each of the three rheumatic diseases reviewed above, and have helped provide a broad contour of the general immune cell landscape in each of these diseases, in both circulation and in afflicted tissues. These studies have also made some inroads into identifying some potentially pathogenic and protective cell types that have been previously underappreciated. A summary of the primary findings reached thus far is included as **Table 1**.

**Table 1:** Summary of human scRNAseq studies in autoimmune rheumatic diseases.

Disease	Reported Findings	Platform	Year	Sample	Patient Number	Cell Count	DOI
**RA**	Atlas of immune cells and stromal cells	Custom Drop-seq	2018	Synovium	5	20,387	10.1038/s41467-017-02659-x
	Two FLS subsets CD55+ in lining layer, CD90+ in sublining						
**RA**	Atlas of immune cells and stromal cells	CEL-seq2	2019	Synovium	51	5,265	10.1038/s41590-019-0378-1
	Two FLS subsets CD4+ PD1hi T peripheral helper						
**RA**	Atlas of immune cells in ACPA+/ACPA- RA	10X Drop-seq	2021	Synovium +Blood	20	206,502	10.1038/s41467-021-25246-7
	Decrease in HLA-DRB5 in B cells in ACPA- Macrophages enriched for CCLs in ACPA-						
**RA**	Focused study of synovial macrophages	10X Drop-seq	2020	Synovium	17	32,141	10.1038/s41591-020-0939-8
	CD206+MerTK+ Macrophages associated with remission Modulation by Gas6 of potential CD90+FLS origin						
**SLE**	Atlas of immune cells	10X Drop-seq	2020	Blood	33	276,000	10.1038/s41590-020-0743-0
	Dominant ISG signature across multiple populations						
**SLE**	Immune cells in limited tissue	CEL-seq2	2019	Urine+Renal biopsy	24	2,881	10.1038/s41590-019-0398-x
	Multiple macrophage populations Tissue-resident BLHLE41+ with enriched ISG signature						
**pSS**	Atlas of immune cells	10X Drop-seq	2021	Blood	5	57,288	10.3389/fimmu.2020.594658
	Increase in CD4+ CTL						

In the case of SLE, which has the largest number of patients sequenced thus far, interferon signaling appears to play the biggest role in differentiating single-cell profiles during both active disease and remission. Multiple immune cell types appear to have skewed differentiation, and this skew extends to tissue infiltrating immune cells. The pervasiveness of this signature raises hope that disruption of the interferon signaling axis, whether *via* monoclonal antibodies or other means, may serve to effectively restrain disease activity in SLE. scRNAseq studies in SLE have thus emphasized a direction for translational research with the establishment of a dominant signaling pathway and the characterization of unique cell subtypes. For instance, if additional mappings are performed to gauge therapeutic response in SLE patients to a particular treatment, it may be possible to utilize these signatures as a key reference point. Assessment of these signatures might also help as a leading indicator to predict relapse or remission. Importantly however, these assessments will require validation in much larger cohorts of patients than those typically sequenced in scRNAseq studies in order to reach sufficient power. As such, developing other assays that can capture the nuanced insights obtained from scRNAseq data will be necessary to further translational research into SLE.

In the context of RA, less is known about the full spectrum of immune cells involved and the essential signaling mechanisms involved. While several sequencing studies of the synovium in RA have been performed, these studies have largely emphasized more local alterations in fibroblast and myeloid signatures in joints. In contrast to SLE, these alterations are not necessarily reflected in peripheral circulation. However, from the clinical correlation analyses reported thus far, it appears that the presence of some of these populations is positively associated with increases in systemic scores of disease activity and inflammation (such as ESR, CRP, and overall DAS28). It is currently unclear how these local changes in one joint may propagate to others, or otherwise influence RA-involved conditions in other tissues. Extensive future investigations are necessary to gain a clearer picture of the relevant molecular mechanisms and immune response patterns in RA, and how these mechanisms might enable autoimmune reactions across the body.

The landscape of gland-infiltrating immune cells in pSS is similarly underexplored. As reviewed above, only a single scRNAseq study of circulating immune cells in pSS has been performed thus far, identifying a potential increase in CD4 CTLs. However, the relationship between circulating phenotypes and glandular damage in pSS remains unclear. Although there have been some indications of interferon signaling playing an important role in pSS based on transcriptome differences relative to healthy controls, it is currently unclear if strong ISG signatures can be found in both circulating and gland-infiltrating immune cell populations on a single-cell level in a manner akin to SLE. Detailed mappings of both peripheral blood and infiltrated glands in pSS patients will be necessary to clarify these and other possibilities.

Besides the information already obtained from atlas studies, scRNAseq also has immense potential for uncovering additional biological insights by being integrated into other forms of omics information. In recent years, other sequencing-based omics technologies have become increasingly mature and are now available through established protocols. In the following section, we highlight single-cell immune repertoire sequencing and spatial transcriptomics as two technologies with particular potential to be applied in assessing rheumatic diseases and answer key questions regarding their pathological progression.

## Challenge 1: Lineage Tracing and Identification of Clonally-Proliferating Autoreactive T and B Cell Populations

Current scRNAseq studies of rheumatic diseases have elucidated a significant number of distinctive T and B cell populations as reviewed above. However, classification of these T and B cells has been driven by transcriptome-level information on expression profiles, and not based on their antigenic specificity, thus neglecting a critical component of T and B cell identity. Antigen specificity of T and B cells is determined by the sequence of unique recognition receptors [T cell receptor (TCR) and B cell receptor, respectively] encoded as specific sequences generated by genetic recombination (56, 57). Unfortunately, in 3’ biased sequencing data, it is not possible to directly recover these sequences. As such, the scRNAseq studies of autoimmune rheumatic diseases have thus far been unable to make direct determinations regarding the clonal status of T and B cells.

Fortunately, new approaches for library construction have made it increasingly possible to capture full-length sequences, including in the TCR and BCR regions. In plate-based workflows, utilization of unique enzymes and/or amplification sequences (such as template-switching oligonucleotides with Moloney murine leukemia virus reverse transcriptase, or not-so-random primers) generate full-length information and capture entire TCR and BCR sequences (58, 59). In droplet-based sequencing, 5’ library construction is now available through commercial platforms, and the addition of a second workflow for targeted amplification of TCR and BCR sequences can capture sequences in the complementarity determining region 3 (CDR3). As such, it is now possible to perform paired single repertoire sequencing along with scRNAseq to simultaneously profile the clonal identity and transcriptome of a given cell.

This advance has opened up new possibilities, as the importance of autoreactive T and B cells clones in mediating rheumatic diseases has long been appreciated. Early studies in animal models raised the hypothesis of there being ‘forbidden clones’ driving autoimmune diseases (60), and subsequent findings in patients have helped to demonstrate the clear and common occurrence of autoreactive clones in rheumatic diseases. For instance, antibodies against double-stranded DNA (dsDNA) and other cell nucleus components (ANA) is a hallmark of SLE (61), while anti-Ro/anti-La (62) and ACPA (63) antibodies are observed at high frequencies in patients with SS and RA, respectively. Positive detection and circulating titers of these autoantibodies is now routinely used as a key parameter during clinical assessments of disease severity. In the context of SLE and SS in particular, deposits of these autoantibodies can often be found in afflicted tissues (particularly in renal glomeruli in SLE and salivary glands in SS) and contribute directly to disease pathology. Local stromal cells, such as salivary epithelial cells, might also promote the survival of these autoantibody-producing B cells as part of a vicious cycle (64). Similarly, autoreactive T cells have been reported for rheumatic diseases, although their presence is not assessed as part of traditional clinical evaluations (65, 66). Autoreactive CD4+T cells can produce large amounts of cytokines to maintain inflammatory environments, while autoreactive CD8+T cells may directly lyse their target cells. These and other ancillary pathogenic characteristics have led to the view that targeting of autoreactive clones may be an efficient therapeutic strategy for rheumatic diseases.

However, it is also important to note that autoreactive T and B cell clones will only represent a small portion of the total immune repertoire of any individual at a given time, particularly in circulation. Furthermore, positive serum detection of autoantibodies has been reported to precede clinical disease in SLE, SS, and RA, and a high percentage of people who do not develop rheumatic diseases may also have circulating autoantibodies (67). In the context of SLE, it has been reported that ANA-reactive B cells can be found among the plasma cells in peripheral circulation of lupus patients, but that these clones may only represent less than 1% of circulating plasma cells. It is unclear at this time whether autoreactive clones will be more highly concentrated in B cells of a particular phenotype/maturation status, or if they will be distributed in line with unrelated clones. It is also unclear if autoreactive clones might have differences in developmental trajectory compared to unrelated counterparts.

Given the fact that patients with rheumatic diseases typically maintain significant circulating titers of autoantibodies, one might assume that autoreactive clones may tend to occupy a higher percentage of class-switched memory and plasma cells which can directly secrete large amounts of antibody. However, several studies that have inspected the proportion of ANA-reactive B cells by stage in SLE patients have found only a surprisingly small increase in circulating ANA-reactive plasma cells compared to controls (68, 69). Similarly, percentages of RF and ACPA B cells may only represent 1% of circulating B cells in patients in RA (70, 71). While a single clone at 1% representation may stand out as being highly expanded in repertoire sequencing analysis, it may not be clearly dominant amongst all clones and be difficult to identify. Furthermore, a single autoantigen may trigger oligoclonal expansion, in which multiple clones expand concurrently, such that no single clone clearly emerges as dominant. As such, it can be surmised that the large majority of B cells reported in scRNAseq studies of peripheral blood do not belong to autoreactive clones and will not produce autoantibodies, and that distinguishing the autoreactive clones may be a difficult endeavor. It may also be possible that the expression profiles and trajectory behavior of autoreactive clones are indistinguishable from that of unrelated clones that have been exposed to the same inflammatory environment.

Alternatively, it is possible that a more precise definition of B cell status derived from scRNAseq, when analyzed together with clonal identity, might help identify subtypes of plasma cells highly enriched for autoreactive clones. Such enrichment may also be more prominent when analyzing a more specific context, such as during clinical remission or abrupt flares. Furthermore, it is critical to remember that B cells can undergo somatic hypermutation and affinity maturation, wherein B cells can reshape their BCR sequence to increase their affinity for a given antigen in germinal centers under T cell help (72). As such, it is possible that many of the apparently non-autoreactive B cells may mature into autoreactive clones once their affinity is raised to a sufficiently high level. Focused evaluation of one particular antigen then, such as dsDNA (73), instead of the broader spectrum of ANAs, may also yield more direct information on specific autoreactive clones. Through currently available computational approaches that can generate B cell clonal lineage trees, it may be possible to gain additional insight into autoreactive clones should enough BCR sequencing and antigen affinity data become available (74).

Since T cells lack the ability to undergo repeated rounds of affinity maturation and class-switch recombination, the TCR sequence of any given clone can be expected to stay stable, allowing for easier analysis. However, due to technical difficulties with generating tetramer libraries against possible autoantigens, even less is known about the behavior and frequencies of autoreactive T cell clones in rheumatic diseases at this time. In the context of pSS, and cloned T cells lines recognizing SSA have been generated (75), and autoreactive Th17 cells producing IL-17 following recognition of a peptide in the M3 muscarinic acetylcholine receptor have also been reported (76). Similarly, a number of studies have remarked that T cells isolated from the synovial fluid of patients with RA show signs of oligoclonal expansion, and may be found at higher frequencies than in peripheral blood, leading to a tempting inference that these expanded clones might be autoreactive (77–79). Some validation studies have also identified specific autoantigens, such as cartilage proteoglycan (80) and citrullinated tenascin-C (81), which may be recognized by these autoreactive clones. Unfortunately, because synovial fluid of RA patients may also contain high concentrations of T cell-recruiting chemokines such as CXCL10 and CCL5, non-autoreactive memory cells expressing cognate chemokine receptors may also migrate to joints.

**Figure 1:**
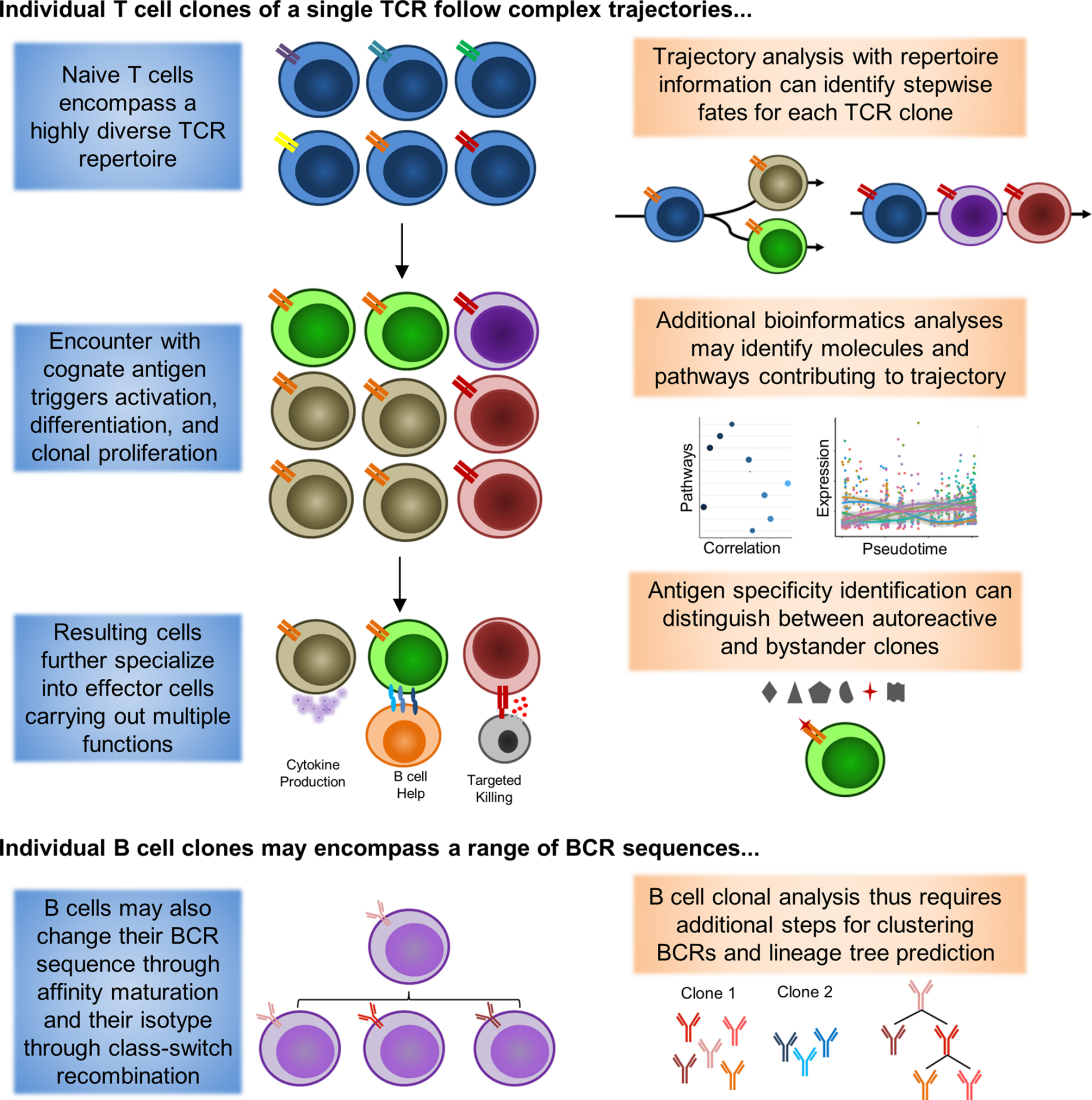
Single-cell Lineage Tracing of Autoreactive T and B cell Clones. Expanded T cell clones emerge from a pool of naive T cells (blue) with similar phenotypes and a highly diverse TCR repertoire (double bars of different colors representing distinct TCRs). Extrapolation from sequencing of peripheral blood has yielded estimates of 10E8 distinct TCRs among naive T cells in circulation alone. Under resting conditions, these naive T cells are expected to only undergo homeostatic proliferation, such that the numbers of cells belonging to a particular clone should not substantially increase. However, when these naive T cells encounter a presented cognate antigen, they can undergo activation, differentiation, and clonal proliferation (orange and red TCRs). As a consequence, formerly naive T cells will adopt novel functional characteristics (different cell colors representing distinct phenotypes). Notably, a single clone can differentiate into multiple different functional states that coexist at the same point in time, a process in which asymmetric cell division has been proposed to be critically involved. Some differentiated characteristics are may be somewhat unique to CD4+ (B cell help) or CD8+ (targeted killing) T cells, although these functions are not absolutely exclusive. Similar to T cells, B cells will also undergo activation/maturation, differentiation, and clonal proliferation from a pool of naive progenitors with high BCR sequence diversity. However, B cells also have the unique ability to undergo somatic hypermutation during the process of affinity maturation to modify their BCR sequence (different BCRs represented by unique, but similar, colors of the immunoglobulin symbol). As a result, a single BCR clone observed in plasmablasts may encompass multiple unique BCR sequences in naive B cells that have subsequently converged. Additional bioinformatics tools for BCR clustering and lineage tree tracing have been developed to help identify clones potentially linked by somatic hypermutation events.

As such, further work will be necessary to distinguish the identities of autoreactive and bystander memory clones. A summary of how inclusion of repertoire information may help guide analysis is depicted in **Figure 1**. Incorporation of TCR and BCR repertoire data into scRNAseq thus offers an exciting new lens for analyzing and understanding the pathogenesis of rheumatic diseases, and may shed light on the true behavior of autoreactive clones in rheumatic diseases.

## Challenge 2: Spatial Mapping of scRNAseq Data and the Tissue Specification of Pathogenic Immune Cell Populations

The recent advent of spatially-aware transcriptomics has made it possible to gain a concurrent understanding of the tissue localization and transcriptomes of individual cells (82). While pioneering studies in spatial transcriptomics were somewhat limited in point resolution to small clusters of cells, novel improvements in barcode generation and seeding have now made single-cell analysis possible. In the context of rheumatic diseases, this technology holds considerable promise for enabling comprehensive analyses of the function and origin of tissue-infiltrating immune cell populations, and unveiling the identity and status of the stromal cells that they interact with. Spatial transcriptomics may prove to be especially useful for elucidating the mechanisms driving multi-organ involvement seen in patients with severe disease.

**Figure 2:**
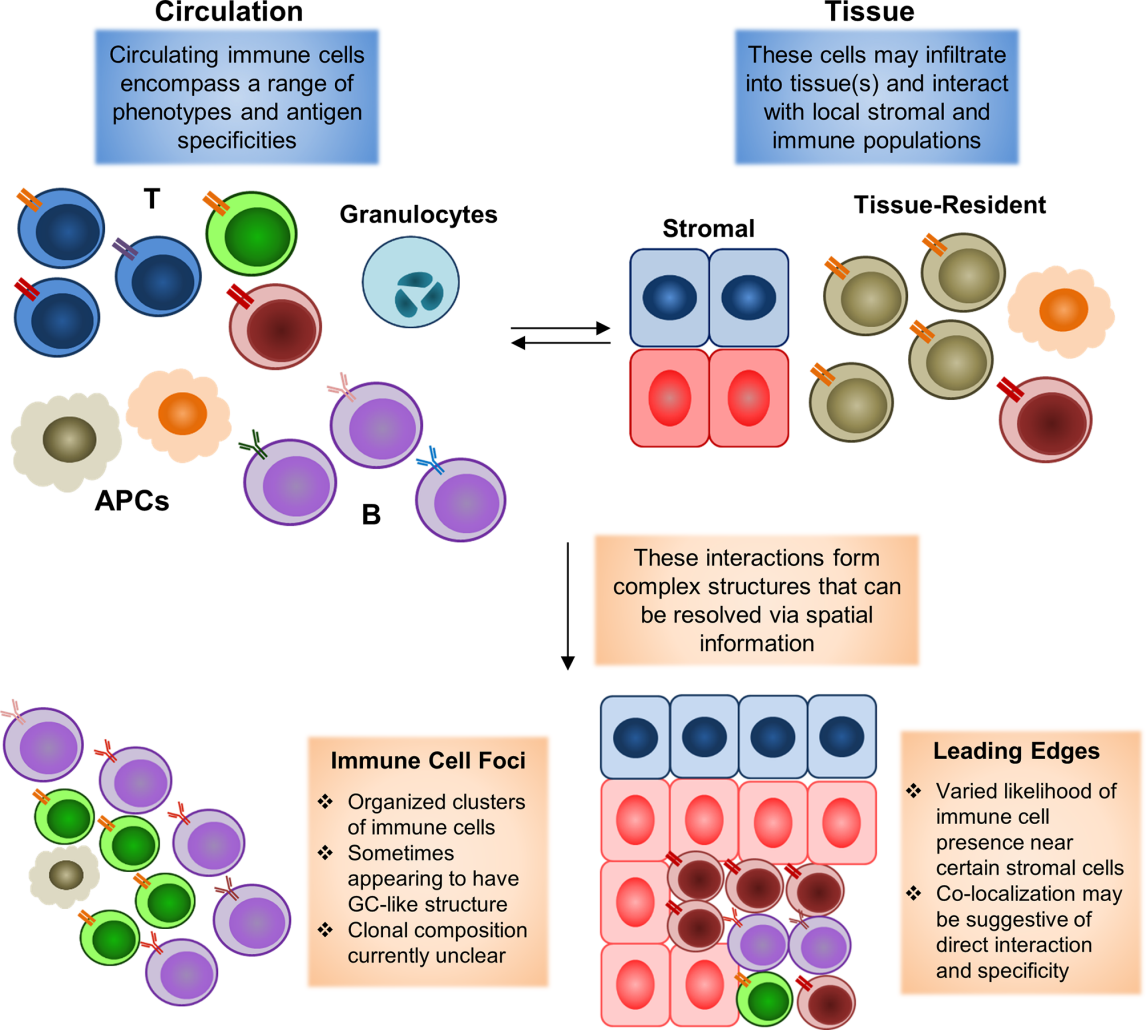
Spatial mapping of tissue-infiltrating immune populations. During an immune reaction, both circulating and tissue-resident cell populations will play significant roles. The formation of an inflammatory environment in tissue with high levels of cytokines can induce rapid recruitment of circulating immune cells. These recruited cells may then infiltrate into the tissue to carry out their disparate functions. These tissue-infiltrating immune cells will also interact with local residential populations of stromal and immune cells. Tissue-resident populations may also egress into circulation to carry out functions elsewhere in the body (with perhaps the most clearly defined example being the migration of tissue-resident dendritic cells bearing antigens into lymph nodes to initiate adaptive immune responses there). Animal model studies have demonstrated that both the recruitment of immune cells from circulation to a specific site and the egress of site-resident populations to distal tissues may begin in a manner of minutes following pathogen exposure. Complex interactions between resident and newly-infiltrating immune cells may then lead to the formation of histologically-observable structures, such as immune cell foci, in which T and B cells typically converge in an ordered manner to form a structure similar to germinal centers observed in secondary lymphoid organs (spleen and lymph nodes). Leading edges of immune cell infiltration may also be observed, in which some types of immune cells appear to be restrained from infiltrating into some portions of a tissue, or otherwise highly enriched near some stromal cell populations, but not others. These edges may form as a result of modulation by the local environment, in the form of inhibitory signals or other factors. Existing histological studies have widely documented the occurrence of immune cell foci in autoimmune rheumatic diseases, with the presence of these foci being a common diagnostic criterion for pSS. Leading edges have more commonly been observed in the context of tumors as a result of spatial transcriptomics analyses, but have yet to be clarified in the rheumatic diseases due to the lack of similar datasets thus far.

For instance, SLE is known to cause systemic manifestations in an extraordinarily wide range of tissues spanning the entire body, and current studies of skin and renal manifestations. Single-cell mapping studies have further demonstrated that significant numbers of ISGhi immune cells may enter into tissue sites. However, current analyses of these infiltrating immune cells have been limited to transcriptome information without spatial location. A spatial mapping might be able to resolve the context in which these immune foci form, as well as the contents of each foci. One question of particular interest is whether all of these foci are indeed dependent on interferon signaling, and which communication signals from surrounding stromal tissue may serve to help/hinder their formation. For instance, immune foci have been found to be preferentially localized near certain types of cells, but not others, in the context of tumors (83, 84), but it is currently unclear if similar biases in cell type distribution can be observed in rheumatic diseases. Resolving these questions, as highlighted in the schematic shown in **Figure 2**, may help answer key questions concerning stromal-immune cell interplay in rheumatic diseases.

As reviewed above, histology-guided analyses in RA have already helped to identify differences in spatial distribution between Thy1+ and CD55+ FLS, and have found preferential accumulation of immune cells near Thy1+ FLS. A preliminary spatial transcriptomic study of RA synovium has also been conducted, and has suggested co-localization between CD8+ T effector memory cells and class-switched B cells, and segregation between T cells and plasma cells (85). Unfortunately, the spatial context of other immune and stromal cell types in synovial tissue and in the joint microenvironment remains unclear. For instance, stromal populations such as articular chondrocytes that maintain the cartilage matrix may also be involved in local inflammatory processes during RA progression (86), and osteoblasts have been shown to be receptive of immune cell stimuli (87). A fuller characterization of the spatiotemporal heterogeneity of these and other stromal cell types on a single-cell level might help to greatly expand current understandings of RA pathogenesis.

Furthermore, spatial analyses may also help to compare newly-infiltrating immune cells with long-lived residential immune cells that can persist in many tissues. For example, the skin under normal conditions is expected to contain large numbers of Langerhans cells and memory T cells. These cells provide an important barrier function as a first line of defense against external pathogens, and have been conditioned to survive and function in the dermal environment. These residential cells can thus be expected to express unique transcriptome profiles that enable local adaptation, and can more readily respond to local insults by virtue of their localization (88). Traditionally, residential populations are expected to react against known pathogens, but autoreactive clones have also been shown to adopt residential characteristics in autoimmune diseases, particularly in the skin (89, 90). However, it is not completely understood how or if the distribution and function of these residential cells may be modified by further infiltration of non-residential immune cells. It is also unclear how cells with elevated ISG signatures may be distributed in the skin, or if immunosuppressant treatment to treat SLE will have a differential impact on these residential populations as compared to newly-infiltrating ISGhi cells as a result of differences in location and drug bioavailability. These results will also have significant ramifications for understanding side effects associated with immunosuppressant use, such as risk of infection. A spatially-guided study in these and other related scenarios may be of great use for answering these questions and clarify the true functional relevance of immune cell structures in afflicted tissues.

## Conclusion

The application of scRNAseq into human rheumatic disease holds great promise for answering foundational questions regarding how autoimmune reactions are instigated and maintained in complex environments, as well as identifying novel targets for therapeutic intervention. Current efforts to map the atlas of immune cell types involved in each disease have already identified a number cell types and pathways as being of notable interest, particularly in the context of SLE. These mapping studies have also identified notable modifications in regional, tissue-resident populations of both immune and non-immune cells as a result of disease progression that point to larger-scale remodeling caused by immune cell activity. If recently emerging technologies for repertoire and spatial mapping can be integrated into scRNAseq studies, a much more complete picture of auotreactive lymphocytes and tissue infiltration patterns can also be obtained. Combination of these assays with other emerging single-cell technologies in each sample, such as ATAC-seq (91) and proteomics (92), might also assist in generating even more comprehensive multi-omics data. Future studies taking advantage of these existing resources and newer technologies may help bring about breakthroughs in treatment for rheumatic diseases.

## Author Contributions

YW and LZ generated the central concepts for the manuscript. ZZ and LC collected and assessed the primary literature in the field and drafted initial figures. ZZ, LC, JL, and GC wrote the initial manuscript. YW, GC, and LZ extensively revised the manuscript. All authors contributed to the article and approved the submitted version.

## Funding

This work was funded by support from the National Natural Science Foundation of China (NSFC 81971546 and 82171787 to LZ, 81971537 to JL), and from the Chongqing International Institute for Immunology (2020YJC06 to LZ).

## Conflict of Interest

The authors declare that the research was conducted in the absence of any commercial or financial relationships that could be construed as a potential conflict of interest.

## Publisher’s Note

All claims expressed in this article are solely those of the authors and do not necessarily represent those of their affiliated organizations, or those of the publisher, the editors and the reviewers. Any product that may be evaluated in this article, or claim that may be made by its manufacturer, is not guaranteed or endorsed by the publisher.
